# Rapamycin adjuvant and exacerbation of severe influenza in an experimental mouse model

**DOI:** 10.1038/s41598-017-04365-6

**Published:** 2017-06-23

**Authors:** Ching-Tai Huang, Chen-Yiu Hung, Tse-Ching Chen, Chun-Yen Lin, Yung-Chang Lin, Chia-Shiang Chang, Yueh-Chia He, Yu-Lin Huang, Avijit Dutta

**Affiliations:** 1 Division of Infectious Diseases, Department of Medicine, Chang Gung Memorial Hospital and College of Medicine, Chang Gung University, Kweishan, 33333 Taoyuan Taiwan; 2 Department of Thoracic Medicine, Chang Gung Memorial Hospital, Kweishan, 33333 Taoyuan Taiwan; 3 Department of Pathology, Chang Gung Memorial Hospital and College of Medicine, Chang Gung University, Kweishan, 33333 Taoyuan Taiwan; 4 Division of Hepatogastroenterology, Department of Medicine, Chang Gung Memorial Hospital and College of Medicine, Chang Gung University, Kweishan, 33333 Taoyuan Taiwan; 5 Division of Hematology and Oncology, Department of Medicine, Chang Gung Memorial Hospital and College of Medicine, Chang Gung University, Kweishan, 33333 Taoyuan Taiwan

## Abstract

Influenza virus infection often causes severe disease and acute respiratory distress syndrome. It is a common belief that overwhelming immune response contributes to the severe illness. Physicians and researchers have put forth immune modulation as salvage therapy for better recovery. However, empiric corticosteroid failed in both humans and animal models. Reported success with Rapamycin in humans prompted a comprehensive animal study and mechanistic dissection. Here we report the effect of Rapamycin alone or in combination with Oseltamivir for severe influenza in BALB/c mice. We found that Rapamycin had no antiviral effect against H1N1, H3N2 and novel-H1N1 influenza viruses *in vitro*. Rapamycin alone aggravated the severe disease of PR8 H1N1 influenza virus infection in mice. Timely Oseltamivir anti-viral therapy abolished the disease. Delayed Oseltamivir treatment could not prevent severe illness and Rapamycin adjuvant was associated with exacerbated disease. Rapamycin adjuvant suppressed influenza hemagglutinin antigen-specific T cell immunity and impaired virus clearance from the lungs. It also resulted in intensified lung pathology with increased intra-alveolar edema and hyaline deposition. Rapamycin may work as the salvage therapy for severe influenza but it is very difficult to define the appropriate window for such treatment to take effect.

## Introduction

Influenza virus poses continuous threats to our society with incessant seasonal infections, epidemics and pandemics (http://www.cdc.gov/flu/). The severity of the disease depends on the interplay between viral virulence and host resistance. The influenza virus circumvents host defenses for establishment of infection with efficient replication^1, 2^. The host immune system, on the other hand, responds to limit the viral spread and works to eradicate the virus^3–7^. Effective host immunity clears the virus without damage to the host resulting from over-reaction. However, inappropriate aggressive host response with excessive inflammatory reaction and lung injury has been correlated with unfavorable prognosis of severe influenza in humans and in several experimental studies^3–10^. Control of inflammation and cytokine storm is a major concern, in addition to eradication of the virus, in management of severe influenza.

Oseltamivir (Tamiflu™) is the major antiviral agent used for people with influenza virus infection. Oseltamivir is most effective when it is taken within the 48 hours from symptom onset^11, 12^. It reduces the duration of symptoms by about one day^13^. There is no solid evidence that it may prevent severe illness^14–16^ (http://www.cdc.gov/mmwr/preview/mmwrhtml/rr6001a1.htm, accessed October 07, 2016). Immune modulation for control of lung inflammation may be of help. Salvage therapy with corticosteroids failed in almost all clinical trials^17–19^. The results with mTOR inhibitors remain ambiguous. Everolimus therapy improved the outcome with a delay of 1.8 days in death and a marginal survival benefit beyond 14 days post-infection in an animal model^20^. There has been a reported success in human H1N1 virus infection with adjuvant Rapamycin in addition to standard Oseltamivir treatment^21^. As Rapamycin impaired virus eradication^20^ and failed to provide any benefit against primary influenza virus infection in another study^22^, a comprehensive animal study with mechanistic dissection is urgently desired^23, 24^.

Here we study the effect of Rapamycin alone or as an adjunct with Oseltamivir in severe influenza through the use of our influenza hemagglutinin (HA) antigen-specific transgenic mouse experimental system. Rapamycin had no antiviral effect against influenza viruses both *in vitro* and *in vivo*. Rapamycin alone aggravated the severe disease of PR8 H1N1influenza virus infection. Timely Oseltamivir anti-viral therapy abolished the disease. Delayed Oseltamivir treatment could not prevent severe illness and additional Rapamycin adjuvant was associated with exacerbated disease. Rapamycin adjuvant suppressed HA-specific T cell immunity and impaired virus clearance from the lungs. It also resulted in intensified pathology of the lungs with increased intra-alveolar edema and hyaline deposition.

## Results

### A mouse model of severe influenza

**Figure 1:**
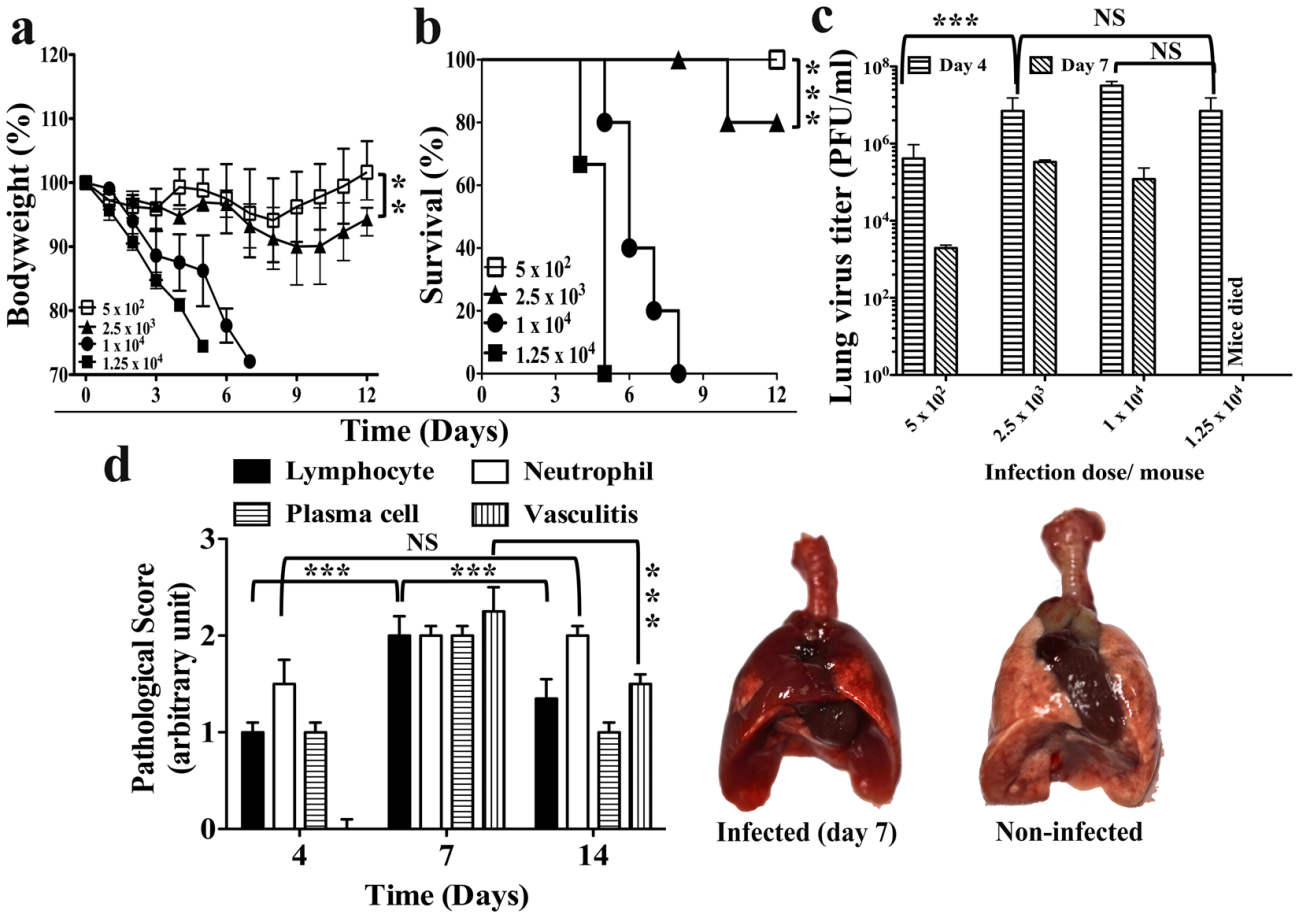
Mouse model of severe influenza virus infection. (**a**) Body weight loss, and (**b**) survival of BALB/c mice following intranasal infection with stated doses of PR8 strain of H1N1 influenza virus. (**c**) Lung virus titer on the stated days post infection with indicated doses of PR8 influenza virus infection in BALB/c mice of same age group. (**d**) Pathological analysis of lung tissue sections (Left panel) and gross lung pathology (Right panel) of BALB/c mice on day 7- post infection following infection with 2.5 × 10^3^ PFU of PR8 influenza virus. Data are representative of at least three similar experiments and presented as mean ± s.d. (***p < 0.0001; **p < 0.001; NS = non-significant, p > 0.05; n = 6/group).

Disease severity varied with the inoculum size of influenza virus infection in BALB/c mice. Intranasal infection with inoculum sizes over 2.5 × 10^3^ plaque-forming units (PFU) of live PR8 H1N1 strain virus resulted in severe disease. With 1.25 × 10^4^ PFU virus, ruffled hair and body weight loss became apparent since day 2- post-infection and the mice succumb to death between day 5- to day 8- post infection (Fig. 1a,b). Infection of 2.5 × 10^3^ PFU virus caused disease of less severity. Disease symptoms started from day 3- post infection. Although there was significant inflammation in the lung, with severe congestion and dense patches on gross appearance (Fig. 1d), the majority of the mice recovered from the disease (Fig. 1a,b,c and d) with virus eradication by day 14- post infection. Infection of 5 × 10^2^ PFU of virus displayed minimal body weight loss only and all mice recovered from the disease (Fig. 1a,b). This mouse model^8, 9, 25^ was used for further investigation for the therapeutic potential of Rapamycin, an inhibitor of mTOR (Supplementary Fig. 1), in severe influenza.

### Oseltamivir effectively prevents disease with early treatment

**Figure 2:**
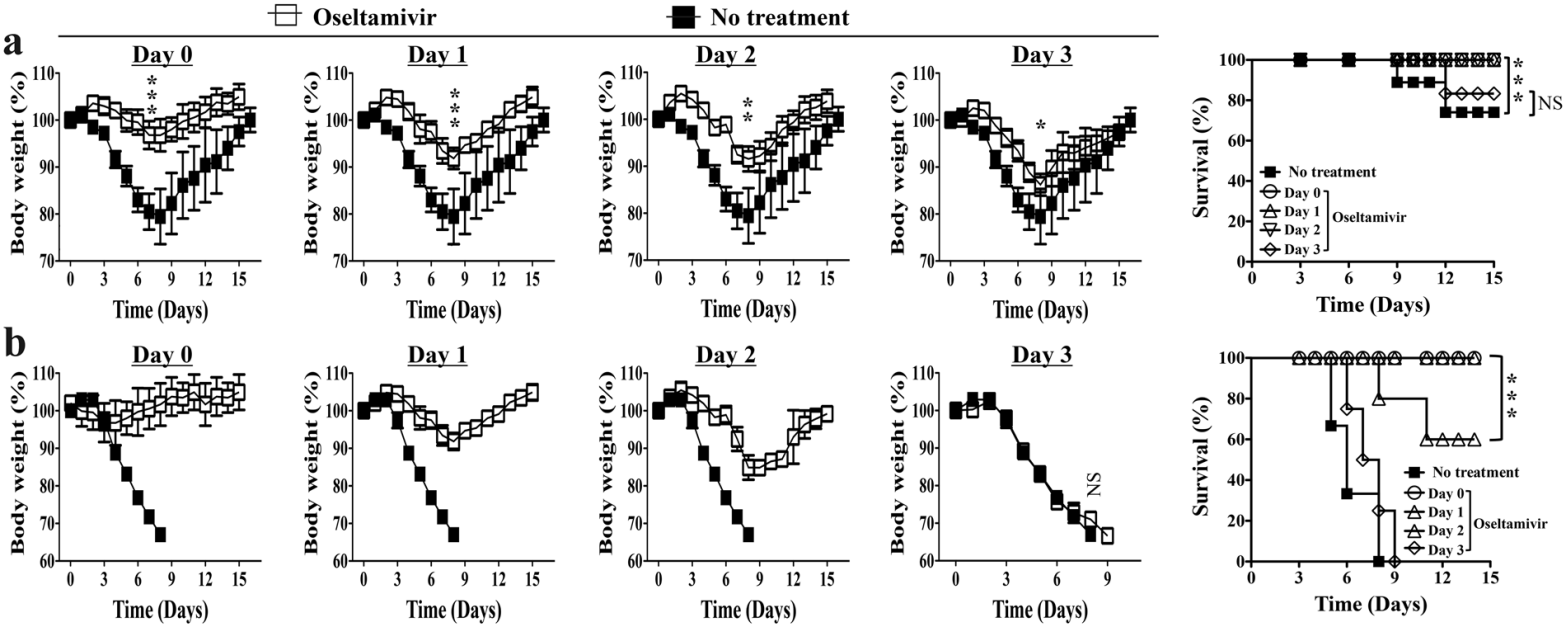
Protective effect of Oseltamivir disappears with delayed treatment onset. Early treatment of Oseltamivir effectively prevented disease of sub-lethal (**a**, inoculum dose = 2.5 × 10^3^ PFU) and lethal (**b**, inoculum dose = 1.25 × 10^4^ PFU) PR8 influenza virus infection in BALB/c mice. The protection decreases with delay of treatment and there was almost no protection with treatment from day 3- post infection. Naïve mice of comparable age were infected with stated inoculum size of PR8 virus. Control untreated mice received daily oral feeding of equal volume phosphate buffered saline (PBS) starting from the stated days post infection of the influenza virus. Data are representative of at least three similar experiments and presented as mean ± s.d. (***p < 0.0001; **p < 0.001; *p < 0.01; NS = non-significant, p > 0.05; n = 6/group).

Oseltamivir was given daily via oral feeding at the dose of 10 mg/kg bodyweight. Treatment abolished the disease for infections with 2.5 × 10^3^ (Fig. 2a) or 1.25 × 10^4^ (Fig. 2b) PFU PR8 influenza virus if started early, either starting on the same day with infection or one day after infection. There was minimal body weight loss and almost all mice survived (Fig. 2). However, the protective effect was lost if treatment was started late. With treatment from day 2- post infection, bodyweight loss was significant and some of the mice died. With further delay of treatment, there was no difference of bodyweight loss between the treated and the untreated control mice (Fig. 2).

### Rapamycin aggravates illness of both mild and severe disease regardless of timing of treatment

**Figure 3:**
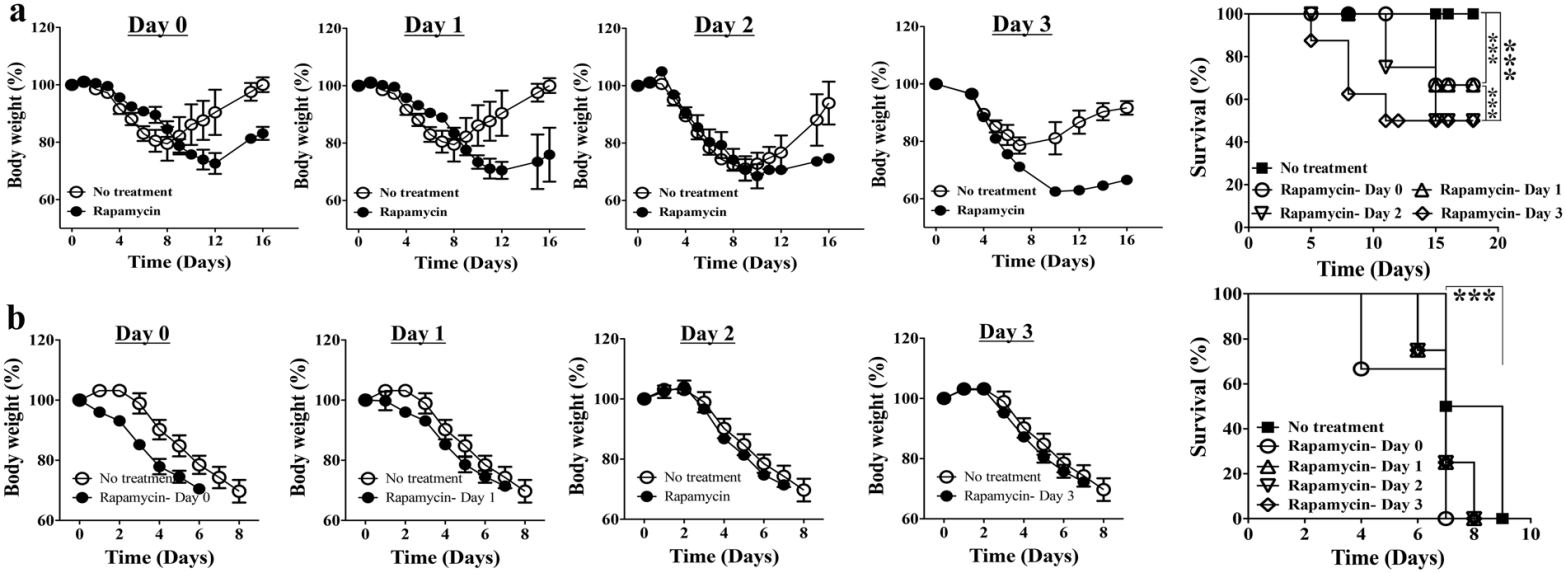
Rapamycin aggravates illness of both mild and severe disease regardless of timing of treatment. Aggravated disease with more bodyweight loss and higher mortality upon daily 3 mg/kg bodyweight Rapamycin treatment compared to that of untreated control BALB/c mice suffering from infection with either (**a**) 2.5 × 10^3^ PFU or (**b**) 1.25 × 10^4^ PFU PR8 influenza virus. Naïve mice of comparable age were infected with stated inoculum size of PR8 virus. Rapamycin treatment was started from the stated days post-infection and control untreated mice received equal volume placebo solvent as daily intraperitoneal injection. Data are representative of at least three similar experiments and presented as mean ± s.d. (***p < 0.0001; n = 6/group).

Rapamycin was given daily with intraperitoneal injection at the dose of 3 mg/kg of bodyweight. Immunosuppressive effects may hurt the mice in the early stage of infection during which effective immunity is desired for virus eradication. We started the timing of treatment starting from either day 0-, day 1-, day 2- or day 3- post infection. Rapamycin caused more severe disease with either 2.5 × 10^3^ or 1.25 × 10^4^ PFU PR8 influenza virus infection, regardless of the timing of treatment. In infection with 2.5 × 10^3^ PFU virus, the body weight loss was more profound in treated mice and some of them died but almost all the untreated mice recovered from illness (Fig. 3a). In infection with 1.25 × 10^4^ PFU virus, both body weight loss and lethality were accelerated with Rapamycin treatment (Fig. 3b).

### Rapamycin does not salvage severe influenza progressive under Oseltamivir

**Figure 4:**
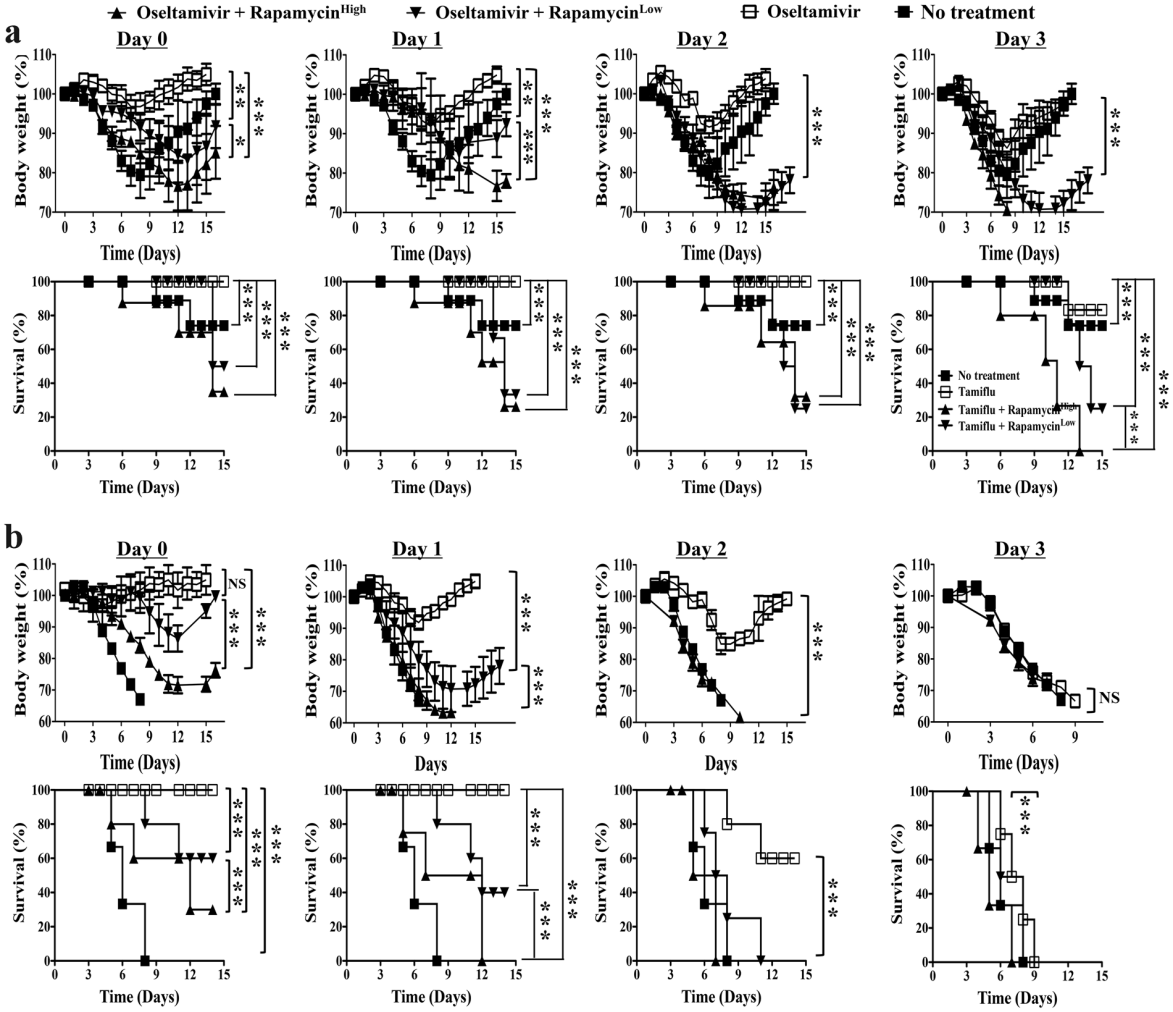
Rapamycin do not salvage severe influenza progressive under Oseltamivir. High (3 mg/kg bodyweight) and low (0.03 mg/kg bodyweight) dose salvage Rapamycin treatment accelerates disease progression and abolishes protective effect of Oseltamivir treatment, regardless of timing of treatment. Naïve BALB/c mice of comparable age were infected with either (**a**) 2.5 × 10^3^ PFU or (**b**) 1.25 × 10^4^ PFU PR8 influenza virus. Rapamycin and Oseltamivir treatment was started from the stated days post infection and control untreated mice received daily intraperitoneal injection of equal volume placebo solvent and oral feeding of phosphate buffered solution (PBS). Mice with Oseltamivir only served as another control group. Data are representative of at least three similar experiments and presented as mean ± s.d. (***p < 0.0001; **p < 0.001; *p < 0.01; NS = non-significant, p > 0.05; n = 6/group).

Patients with suspected influenza may be prescribed Oseltamivir immediately upon presentation to medical attention. Even with this practice, the administration of Oseltamivir was delayed to at least a couple of days after they caught the infection. The delay in severe influenza was even more substantial^11–16^. We tested the outcome with Rapamycin as a salvage measure for severe influenza progressive under Oseltamivir. Mice were infected with 2.5 × 10^3^ or 1.25 × 10^4^ PFU PR8 strain of H1N1 influenza virus and treated daily with the combination of Oseltamivir (10 mg/Kg bodyweight) and high or low dose of Rapamycin (3 mg/Kg or 0.03 mg/Kg respectively) starting from day 0-, day 1-, day 2- or day 3- post infection. Infected mice with no treatment or with treatment of Oseltamivir alone (10 mg/Kg) served as the controls. For Oseltamivir only, effective prevention was compromised with delayed treatment starting from day 3- post infection. About 20% of the mice succumbed to death following infection of 2.5 × 10^3^ PFU virus (Fig. 4a). There was no protection at all following infection of 1.25 × 10^4^ PFU virus (Fig. 4b). Adjuvant Rapamycin aggravated the severity of disease with more loss of weight and a mortality of around 80% following infection of 2.5 × 10^3^ PFU virus (Fig. 4a). Adjuvant Rapamycin not only eliminated the marginal prevention by Oseltamivir but also accelerated disease progression, making it worse than the untreated control mice with infection of higher dose (1.25 × 10^4^ PFU) virus. Mice treated with neither Oseltamivir nor Rapamycin died by day 7- to day 9- post infection. Mice that received Adjuvant Rapamycin died even earlier than the untreated mice. With a Oseltamivir and Rapamycin combination starting earlier than day 3- post infection, the adverse results of Rapamycin adjuvant were more evident. Although they survived better than the untreated control mice, but they always lost more weight and suffered from higher mortality than the Oseltamivir treated mice that did not receive Rapamycin adjuvant (Fig. 4b).

### Rapamycin salvage impairs virus clearance with suppressed antigen-specific T cell immunity

Why did Rapamycin salvage result in inferior outcome in mice with severe influenza? To answer this we further studied anti-viral effects *in vitro* as well as *in vivo* viral clearance, antigen-specific T cell immunity and inflammation in the lungs. Rapamycin, up to a concentration of 200μM, did not display any *in vitro* anti-viral effect on PR8 (A/PR/8/34) H1N1, WSN (A/WSN/1933) H1N1, 3446 (A/TW/3446/02) H3N2, a clinical isolate of novel-H1N1, and influenza B virus with plaque reduction assay in MDCK cell culture (data not shown). On the contrary, Oseltamivir at 10 μM suppressed more than 95% of plaque formation by all these strains. For *in vivo* studies, BALB/c mice were infected with 2.5 × 10^3^ PFU of PR8 virus. Naïve influenza HA (Hemagglutinin) antigen-specific CD4+ T cells were adoptively transferred on the same day of infection. Mice received daily treatment of Oseltamivir (10 mg/Kg bodyweight) only or a combination of Oseltamivir (10 mg/kg bodyweight) and a high (3 mg/kg bodyweight) or a low (0.03 mg/kg bodyweight) dose Rapamycin starting from day 2- post infection.

**Figure 5:**
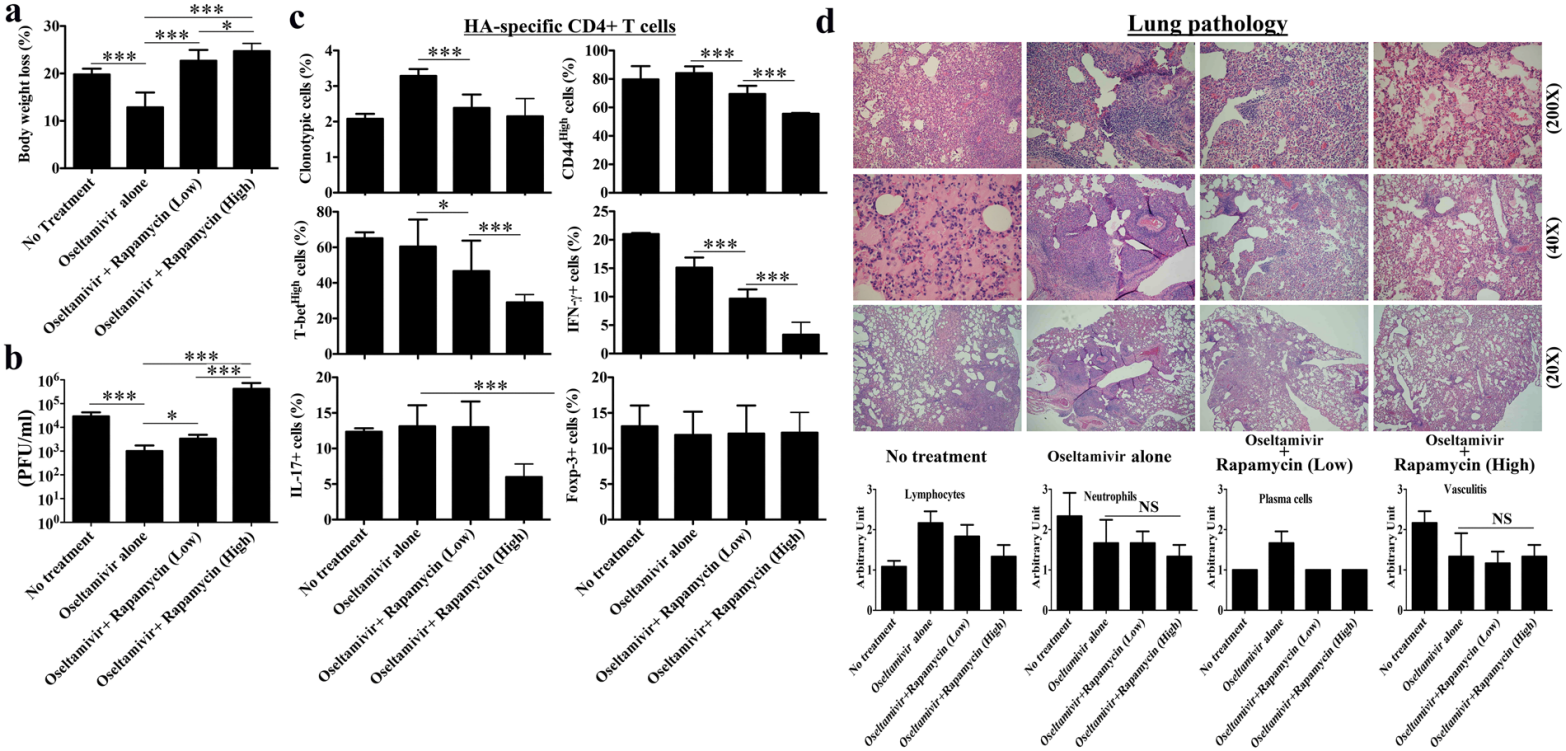
Rapamycin salvage impairs virus clearance with suppressed antigen-specific T cell immunity and aggravates overall lung inflammation with severe intra-alveolar edema and hyaline deposition. Naïve BALB/c mice of comparable age received adoptive transfer of 2.5 × 10^6^ naïve HA-specific 6.5 CD4+ T cells with infection of 2.5 × 10^3^ PFU PR8 virus. Rapamycin and Oseltamivir treatment was started from day 2- post infection and control untreated mice received daily intraperitoneal injection of equal volume placebo solvent and oral feeding of phosphate buffered solution (PBS). Mice with Oseltamivir only served as another control group. On day 7- post infection, compared to untreated and Oseltamivir only mice, Rapamycin salvage treatment caused (**a**) more body weight loss in mice with (**b**) impaired virus clearance in the lungs. Antigen-specific CD4+ T cell immunity was attenuated with low activation status of the stated immune parameters as well (**c**). However, the percentage of Foxp-3+ regulatory T cells in the lungs was comparable among all groups of mice. Histological analysis of lung sections revealed severe hyaline deposition and edema with Rapamycin salvage treatment. Besides less lymphocyte infiltration with Rapamycin salvage, infiltration of neutrophil and plasma cells were comparable among all groups of mice (**d**). All the observed effects of Rapamycin salvage were higher with high dose compared to that with low dose. Data are representative of at least three similar experiments and presented as mean ± s.d. (***p < 0.0001; *p < 0.01; NS = non-significant, p > 0.05; n = 6/group).

Oseltamivir treatment ameliorated the illness with constrained body weight loss compared to mice with no treatment (Fig. 5a). Oseltamivir also enhanced virus clearance by day 7- post infection (Fig. 5b). Rapamycin combination eliminated the protective effect by Oseltamivir. Low dose Rapamycin resulted in more body weight loss and more virus in the lung. The disease was further exacerbated with high dose Rapamycin (Fig. 5a,b).

Oseltamivir treatment resulted in increased infiltration of antigen-specific CD4+ T cells in the lungs on day 7- post infection (Fig. 5c), although the proportions of CD 44^High^ and T-bet^High^ activated Th1 cells and effector cytokine IFN-γ and IL-17 secreting cells of HA-specific CD4+ T cells were comparable to the untreated control mice (Fig. 5c). Rapamycin adjuvant suppressed antigen-specific immunity in the lungs. The clonotypic percentage of HA-specific CD4+ T cells, their activated Th1subsets of cells, and effector cytokine IFN-γ and IL-17 secreting cells among them were all lower than mice with Oseltamivir alone. The suppression by high dose Rapamycin was more than that by low dose Rapamycin (Fig. 5c). However, suppression of HA-specific CD4+ T cell immunity with Rapamycin was not associated with increased regulatory T cells. Percentages of Foxp-3+ HA-specific CD4+ T cells in the lungs were comparable among the mice with no treatment, with Oseltamivir only and with combinations of Oseltamivir and Rapamycin on day 7- post infection (Fig. 5c). However, there was no significant difference in the level of virus neutralizing antibodies among all these groups as tested by plaque reduction assay with serum on day 7- post infection (data not shown).

Despite suppressed antigen-specific CD4+ T cell immunity, the overall lung inflammation was aggravated with the Rapamycin adjuvant. Pathology examination revealed severe intra-alveolar edema and hyaline deposition in the lung by day 7- post infection (Fig. 5d). Although lymphocyte or plasma cell infiltration was lower, the neutrophil infiltration and vasculitis were comparable between mice with treatment of Oseltamivir Rapamycin combination and with treatment of Oseltamivir only (Fig. 5d).

## Discussion

Overwhelming immune response with cytokine storm has been postulated as a major cause of severe influenza and influenza mortality in humans^3–7^. Outcome of severe influenza is dismayed with antiviral agents in addition to optimized supportive care. Immune modulation is a rational approach for the treatment of severe influenza. Negative outcome with broad-spectrum immune suppressants mandates more mechanistic dissection. In this study, we tried to evaluate the therapeutic potential of Rapamycin as a salvage adjuvant for severe influenza progressive under Oseltamivir in a mouse experimental system.

There is no data that definitely proved that Oseltamivir prevents severe influenza in humans. This effect is very clear in mouse models, including ours and many others in literature, if Oseltamivir was taken in time. Mice suffer from progressive severe illness if the treatment starts from day 2- or even later post infection. This fact may explain why prophylactic Oseltamivir is very effective in prevention^26–30^. In prophylaxis, the infected patients get Oseltamivir before, right upon, or very soon after infection. With regards to treatment, it is very difficult for patients to get Oseltamivir in time, i.e., within 48 hours after infection. The delay in severe influenza is even more significant for a number of reasons^31, 32^. In the real world, it is not a surprise that the protective effect with Oseltamivir is very limited. It may reduce the duration of illness by only 24 hours in mild influenza and has no significant effect in severe influenza.

With the suboptimal results Oseltamivir confers, other treatment modalities are urgently desired, especially with modulation of the immune response. Global suppression with corticosteroid never demonstrates any benefit. The results with mTOR inhibitors remain ambiguous. Everolimus therapy improved the outcome with a delay of 1.8 days in death and a marginal survival benefit beyond 14 days post-infection in an animal model^20^. There has been a reported success in human H1N1 virus infection with adjuvant Rapamycin in addition to standard Oseltamivir treatment^21^. As Rapamycin impaired virus eradication^20^ and failed in other studies^22^, we proposed a comprehensive animal study with mechanistic dissection for Rapamycin as the salvage therapy in severe influenza.

We failed to demonstrate how the Rapamycin salvage might take effect, with adjustment of all confounding factors including the inoculum size of the influenza virus, the doses of Rapamycin and the timing of Rapamycin treatment. It is very difficult to define the appropriate window for such treatment to work in our animal model. Rapamycin indeed revealed a detrimental effect on anti-influenza immunity. The influenza antigen-specific immunity was impaired, as represented by the attenuated HA-specific CD4+ T cell responses. With compromised antigen-specific immunity, the capacity for virus eradication is reduced with more remnant virus in the lung compared to control mice with no Rapamycin salvage. On the other hand, the inflammation in the lung is not diminished with Rapamycin salvage. The lymphocyte infiltration in the lung was reduced, but the immune cells of antigen-nonspecific immunity was increased with significantly more damage to the ventilation unit alveoli with hyaline deposition in the air space. The mice then suffered from higher severity of illness and mortality. This increased inflammation may be triggered by the virus the lungs.

The lessons we learned from our studies here is that the capacity for viral eradication should not be compromised with the immune modulation to attenuate the inflammation. There should be a window in which the immune response is no longer needed for virus eradication, and Rapamycin salvage might be effective in that specific window. Even if this is theoretically possible, it will probably rarely work in patients with severe influenza, as patients with severe influenza are more likely to be in a variety of windows and never of a homogenous population.

## Methods

### Mice and influenza virus

All mice are of BALB/c background. The TCR-transgenic mouse line 6.5 is of Thy-1.1/1.1 genetic background and expresses a TCR that recognizes an I-E^d^-restricted HA epitope (^110^SFERFEIFPKE^120^). Wild type BALB/c mice were purchased from National Animal Laboratory Center, Taiwan. All mice were kept in individually ventilated cages within a specific pathogen-free environment and used for experiments between the ages of 8–24 weeks. All experiments involving the use of mice were performed in accordance with protocols approved by the “Animal Care and Use Committee” of Chang Gung Memorial Hospital (Approval Numbers: 2013121814; 2015121203). The committee recognizes that all the experiments involving the use of mice were carried out in strict accordance with the Animal Protection Law by the Council of Agriculture, Executive Yuan, Taiwan, R.O.C., and the guideline as shown in the guide for the Care and Use of Laboratory Animals as promulgated by the Institute of Laboratory Animal Resources, National Research Council, U.S.A.

The PR8 (A/PR/8/34) strain of influenza virus was produced in the allantoic fluid of 10-day-old embryonated chicken eggs and characterized by a core facility at Chang Gung University, Taiwan. The H3N2 and novel-H1N1 influenza viruses were kindly provided by Prof. Shin-Ru Shih of Chang Gung University, Taiwan. Mice were inoculated via intranasal route during light isoflurane (Aesica Queenborough Ltd., Kent, UK) anesthesia with indicated plaque-forming units (PFUs) of virus in 50 μl of PBS^8, 9, 25^.

### Rapamycin and Oseltamivir treatment

Rapamycin (Cat #R5000, LC Laboratories, MA, USA) powder was dissolved in DMSO (Sigma) and aliquots were preserved in −80 °C until use. Aliquots were first diluted 1:1 with Cremophor (Cat# C-5135, Sigma) and subsequent dilutions were prepared in phosphate buffer solution (PBS) for experimental use. An appropriate concentration of the solution was directly added to the culture for assessing anti-viral activity *in vitro* or injected into the peritoneal cavity of mice to study effects *in vivo*. Oseltamivir (Tamiflu, 75 mg Oseltamivir/capsule, Roche, Switzerland) was suspended in PBS to achieve desired concentration. The solution was directly added to the culture for assessing anti-viral activity *in vitro* or fed orally to mice for studying effects *in vivo*.

### Neutralizing antibody level in sera and lung lysates

The functional neutralizing antibody against influenza was quantified by plaque reduction assay as described in our previous publication^25^. In brief, sera or lung lysates from mice were collected on day 7- post infection. The sera or lung lysates were heat inactivated for 30 minutes at 56 °C to de-complement. 200 PFU of live virus was allowed to infect monolayers of DMEM cells in the presence or absence of de-complemented sera with specified dilutions for one hour at 35 °C. Motility of virus was blocked with the use of agarose. Reduction of the plaques due to inhibition by anti-influenza antibody in the sera or lung lysates was counted manually after 72 hours of incubation at 35 °C.

### Adoptive transfer

Clonotypic HA-specific CD4+ TCR transgenic T cells were prepared from pooled spleen and lymph nodes of 6.5 transgenic mice. Clonotypic percentage was determined by flow cytometry analysis. The naïve phenotype was confirmed by profiles of activation markers CD44 and CD62L. 6.5 CD4+ T cells (2.5 × 10^6^) were injected through the tail veins of the recipient mice in 0.2 ml of Hank’s Balanced Salt Solution (HBSS)^8, 9, 25^.

### Flow cytometry

Cell suspensions were incubated on ice with saturated concentrations of fluorochrome-labeled mAbs in FACS buffer (PBS plus 0.5% BSA and 0.02% NaN3) as recommended by the manufacturers. Donor T cells were identified using mAbs: biotin conjugated anti-clonotypic 6.5 TCR (provided by H. Von Boehmer), avidin-PE (A1204, Caltag, Burlingame, CA), or Streptavidin-APC (SA 1005, Caltag, Burlingame, CA) and PerCP or FITC conjugated anti-CD4 (553052 or 553047, BD Biosciences). All fluorochrome-conjugated antibodies were from BD Biosciences. Cells were acquired on FACS CALIBUR and analysis was performed using CellQuest Pro (BD Biosciences) or FlowJo (Tree Star, Inc.) software.

### Intracellular cytokine staining

Single cell suspensions (5–10 × 10^6^ cells/well in 24 well plates) from the harvested organs were re-stimulated for 5 hours with 100 μg/ml MHC class-II restricted HA peptide (^110^SFERFEIFPKE^120^) in the presence of 5 μg/ml Brefeldin A (Sigma-Aldrich). The concentration of Brefeldin-A was maintained throughout intracellular cytokine staining. Re-stimulated cells were surface stained with anti-6.5 and anti-CD4 as described above, fixed in IC fixation buffer (00–8222, eBiosciences), washed, and stained in permeabilization buffer (00–8333, eBiosciences) containing fluorochrome conjugated antibodies against target cytokines. For Foxp3 (11–5773, eBiosciences) and T-bet, cells were surface stained, fixed, and permeabilized in fixation/permeabilization buffer (00–5523, eBiosciences), washed, and stained in the permeabilization buffer (00–8333, eBiosciences) containing fluorochrome conjugated antibodies. In some experiments, cells were analyzed *ex vivo* and stained without re-stimulation^8, 9, 25^.

### Plaque assay for virus titer

We measured live influenza virus titer in the lungs of infected mice using a modified Madin-Darby canine kidney cell (MDCK; The American Type Culture Collection) plaque assay^8, 9, 25^. The lungs were collected at the indicated times, one whole lung in 1 ml DMEM, snap frozen in liquid nitrogen, and stored at −80 °C until analyzed. Ten-fold dilutions of the lung tissue homogenates were prepared in DMEM supplemented with 10% FCS, Antibiotic-Antimycotic (15240–062, GIBCO BRL), and 0.00025% Trypsin. A total of 500 μl of each dilution was added to confluent monolayers of MDCK cells in 6 well plates and incubated for 1 hour at 35 °C with 5% CO_2_. Each well of the culture received 2 ml of an agar overlay (0.3%) and incubated for 3 days. Cells were fixed with 10% formalin, agar overlay was removed, and fixed monolayers were stained by crystal violet (0.02% in 2% ethanol). The results were presented as plaque forming unit (PFU)/ml = (mean number of plaques × 2) × (1/dilution factor).

### Histopathology

The lungs from experimental mice were harvested on days as indicated, and each lung was fixed with 2 ml of neutral buffered formalin solution (10%). Following fixation, the lungs were embedded in paraffin and 5 μm sections were cut. Sections were stained with hematoxylin and eosin (H&E) and scored blindly. The infiltration of inflammatory cells, including lymphocytes, neutrophils, and plasma cells, were separately scored by pathologist as negative, 1^+^, 2^+^, or 3^+^ according to the density of infiltration. Vasculitis and fibrosis were also scored as negative, 1^+^, 2^+^, or 3^+^ based on the severity^8, 9, 25^. Overall inflammation in the lungs was represented by the average of these scores.

### Statistical analyses

Data represented as mean ± S.D. We used Graph Pad Prism version 5 for Student’s t-test analyses. We considered *p* values < 0.05 as significant.

## Electronic supplementary material

Supplementary Information
