# Supplementary material for: Rapamycin adjuvant and exacerbation of severe influenza in an experimental mouse model
Source: Sci Rep. 2017 Jun 23;7:4136. doi: 10.1038/s41598-017-04365-6 (PMC5482837; doi:10.1038/s41598-017-04365-6)
Supplement: Supplementary file 1 — Supplementary Information [file 41598_2017_4365_MOESM1_ESM.pdf]

Rapamycin adjuvant and exacerbation of severe influenza in an experimental mouse model

Ching-Tai Huang; Chen-Yiu Hung; Tse-Ching Chen; Chun-Yen Lin; Yung-Chang Lin; Chia-Shiang Chang; Yueh-Chia He; Yu-Lin Huang; Avijit Dutta

Supplementary Figures

Supplementary Figure 1

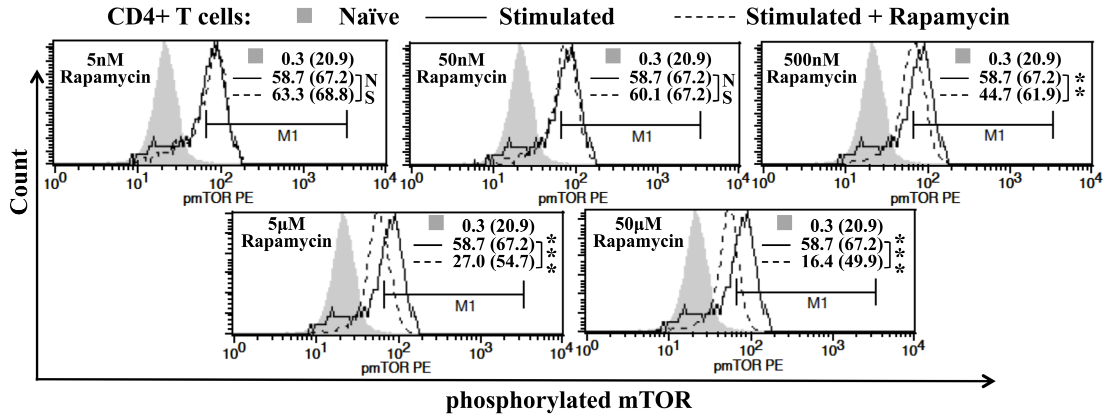

Rapamycin dose-response functionality of mTOR inhibition: The shaded area is the baseline of activated m-TOR in naïve cells. The continuous line indicated the increased level of activated m-TOR with PMA+ionomycin stimulation. The dotted line in each overlay histogram is the level of activated m-TOR with PMA+ionomycin (25 and 500 ng/ml; Sigma) stimulation in presence of the indicated concentration of Rapamycin. Numbers are the percentages of CD4+ T cells in M1 gate and mean fluorescence intensity (MFI) of total CD4+ T cells (in parentheses). Naïve splenocytes from non-infected wild type BALB/c mice were stimulated for 30 minutes, stained for CD4 and intracellular phospho-mTOR (S2448), acquired through BD FACSCalibur, and analyzed with CellQuest Pro (BD Biosciences). Data are representative of at least three similar experiments in duplicates.
